# Effect of Sulfate Concentration on Chloride Diffusion of Concrete under Cyclic Load

**DOI:** 10.3390/ma15062036

**Published:** 2022-03-10

**Authors:** Demei Yu, Chao Feng, Tengfei Fu, Aiqin Shen

**Affiliations:** 1 College of Transportation and Civil Engineering, Fujian Agriculture and Forestry University, Fuzhou 350108, China; yudemei0826@fafu.edu.cn (D.Y.); fchao1227@fafu.edu.cn (C.F.); 2 School of Highway, Chang’an University, Xi’an 710064, China

**Keywords:** concrete, dynamic flexural loading, cyclic, sulfate, chloride, ion transport, coupling function

## Abstract

The existence of chloride ions, sulfate ions, and vehicle dynamic loads may lead to a shortened service life and premature failure of the road and bridge structures in northwestern China. Immersed in a dual-salt solution while simultaneously applying cyclic flexural loads, the free chloride ion concentration and erosion depth in concrete specimens were measured. The influence of the sulfate concentration on the apparent surface chloride concentration (*C*_s_) and apparent diffusion coefficient (*D*_app_) was studied. An exponential model was used to fit the *C*_s_, and the influence of sulfate concentration on the *C*_s_ was analyzed. The result showed that cyclic loading and solution concentration were two primary factors affecting chloride diffusion. Meanwhile, compared with the emersion conditions, dynamic loading would induce significantly accelerated chloride ion penetration. Under the coupling effect of sulfate and dynamic loading, as the sulfate concentration increased, the chloride ion concentration and erosion depth were both decreased. The existence of sulfate ions improved the chloride ion penetration resistance of concrete. The results provide insight in designing concrete in regions where multiple salt ingression (sulfate and chloride) is a major durability issue of the structures.

## 1. Introduction

The existence of chloride ions and sulfate ions in salt lakes and saline soils in northwestern China leads to the corrosion and deterioration of road and bridge structures. For structures in the north region, the large amount of usage of deicing salts and the existence of dynamic vehicle loads in winter aggravate the structural damage, which, in turn, influence the internal pores and microcracks of concrete, further affects the transport behavior of chloride ions in concrete [1,2], and induces corrosion issues of the reinforcing steels during the regular service life [3].

Most previous research has been focused on the influence of stress level on chloride ion transport in existing concrete structures. Different stress levels have different influences on the chloride ion permeability of cyclic, compressively loaded concrete [4]. Wang et al. studied the water absorption rate of concrete after a compressive cyclic load and found that the accumulated water content increased with the increase of load cycles and stress level [5]. Choinska et al. found that the permeability decreased slightly at a 20% stress level of cyclic compression load, then reached the lowest at a 50–60% level, and increased significantly at an 80% stress level [6]. A similar trend was also reported by Lee et al. [7]. Zhang et al. found that the 28 d diffusion coefficient increased under repeated axial compression at 40% and 80% stress levels [8,9]. The influence of the compression load on the chloride ion diffusion coefficient of ordinary concrete is more manifested than high performance concrete [10].

Flexural loads have different influences on the chloride ion transport performance of concrete [10]. Hrabova et al. studied reinforced beams under a long-term static loading state (three-point bending), coupled with a chloride ingress, and proposed potential limit states in a sustainability design [11]. Gowripalan et al. concluded that the chloride ion erosion rate in the tension zone is higher than that in the compression zone under a flexural load [12]. The presence of flexural loading increased the one-dimensional and two-dimensional chloride ion diffusion coefficients of concrete [13]. The influence of dynamic bending loads on the chloride ion transport behavior of concrete has been extensively investigated. Under the coupling action of flexural cyclic loads and chloride ions, the service life of concrete structures will be greatly shortened [14]. The chloride ion erosion of concrete under the dynamic flexural cyclic load was more serious than that under the static load and increases sharply with the increase of the dynamic bending stress level [15,16]. Jaffer et al. found that the reinforcement corrosion products at the aggregate-paste interface under the dynamic load were larger than those under the static load [17]. The existence of the load will also affect the microstructure of the concrete. Cabeza et al. found the change of the microstructure caused by the load affects the ion transport performance [18]. Gontar et al. reported that cracking due to fatigue load could adversely affect both mechanical properties and diffusive behavior [19]. Qi et al. obtained that the generation and propagation of microcracks under the bending load led to the increase of chloride content in recycled aggregate concrete [20]. 

As it is now, there are a few studies that applied cyclic loading coupled with multiple salt ingress. Wang et al. investigated the transport properties of concrete under the cyclic load and environmental factors [21]. However, the salt ingress soaking was done after the cyclic load was applied on samples, which usually cannot reflect the authentic ingression conditions. In this study, a patented testing scheme was designed and customized to perform cyclic loading and salt ingress simultaneously. The influences of the bending cyclic load and sulfate on chloride ion transport behavior was studied by analyzing chloride ion distribution, erosion depth, apparent surface chloride ion concentration, and apparent diffusion coefficient. The results will provide insight in designing concrete in regions where multiple salt ingression (sulfate and chloride) is a major durability issue of the structures. 

## 2. Materials and Methods

### 2.1. Materials

P.O 42.5 cement was purchased from the Jidong Cement Company (Xi’an, China). The chemical compositions are shown in Table 1. 

Concrete proportion and flexural strength are presented in Table 2. The fineness modulus of the river sand was 2.82. The coarse aggregate with continuous gradation was crushed by Shaanxi limestone, the size range was 5–20 mm, and the apparent density was 2710 kg/m^3^. The clean tap water was used as mixing water. The sulfate ion in water reducer was less than 0.01% by mass; thus, the sulfate ion in admixtures can be neglected.

To mix the concrete, aggregates were added to the mixer and dry mixed for 60 s, and then, cement was added during the next 30 s. Then water and superplasticizer were added, respectively, during the next 30 s and mixed for another 90 s before the fresh concrete was poured into 100 mm × 100 mm × 400 mm molds. All concrete specimens were kept at room temperature for 24 h and then demolded and cured in a standard curing room with 20 ± 2 °C and >95% RH for 28 d. Before each cyclic loading, specimens were placed in an oven at 50 °C for 24 h and then cooled to room temperature. The purpose was to drive out excessive free water in the specimens and to better mimic the field condition.

### 2.2. Dynamic Flexural Loading and Erosion Setup

**Figure 1:**
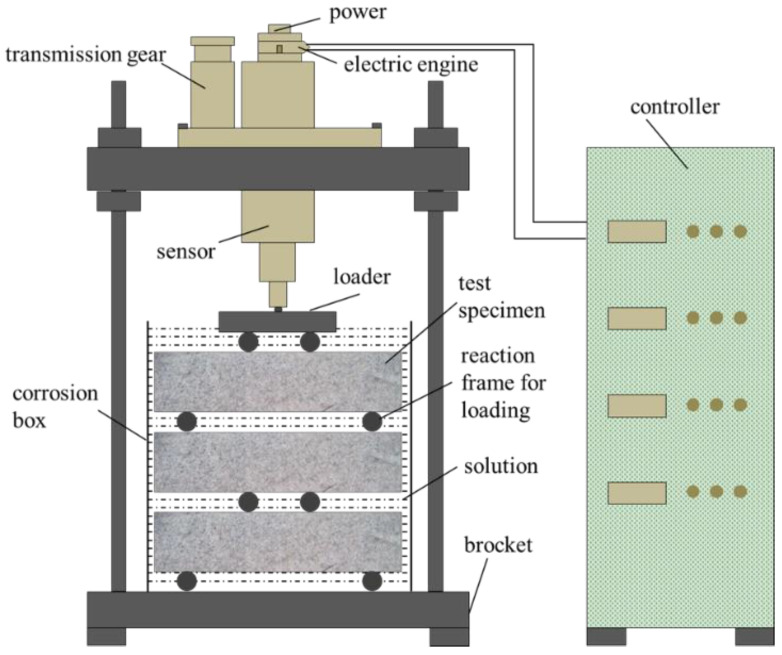
Scheme of the flexural loading device with the erosion chamber.

The 28 d flexural strength was used as the initial strength to calculate the loading stress level. The mechanical erosion cyclic test device is shown in Figure 1. The loading system was customized, based on the universal mechanical testing machine, and patented (Chinese patent number: CN201819856U). 

**Figure 2:**
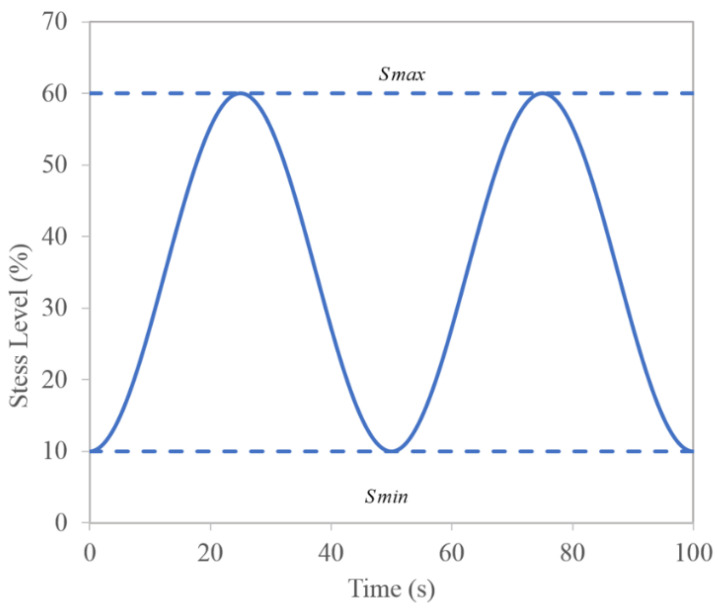
Loading diagram.

The specimens with five surfaces were sealed with epoxy and plastic wrap; one side was selected as the exposed surface to bear up the erosive solution as well as the cyclic loading. The treated specimens were immersed in a container filled with a prepared erosive solution (8% NaCl, 8% NaCl + 5% Na_2_SO_4_, 8% NaCl + 10% Na_2_SO_4_). The cyclic loading was a sine wave function increasing from a stress level of 10% (*S*_min_) to 60% (*S*_max_), then dropping back to 10% (as shown in Figure 2), with a 50 s full cycle (0.02 Hz). More details can be found in the literature [22]. This cycle continued throughout the salt ingression. The loading durations were 20 d, 40 d, 60 d, 80 d, and 100 d, respectively. Table 3 shows the experimental program of the cyclic loading condition and salt ingression.

The erosion cyclic specimens were removed and dried after the specified erosion age. The room temperature (20 ± 3 °C) was controlled by air conditioning to avoid the influence of temperature effect.

### 2.3. Chloride Transport Behavior Evaluation of Concrete

#### 2.3.1. Free Chloride Concentration

The powder was collected from the exposed surface by a drill with a diameter of 5 mm. The sampling depth was 3 mm, 6 mm, 9 mm, and 12 mm until it reached 30 mm. The powder was sieved through a No. 100 (with 0.15 mm opening) sieve. The free chloride concentration was evaluated according to ASTM D512-12 [23]. It should be noted that when preparing samples for calculating chloride concentration (% concrete), large aggregate particles were discarded to reduce the grinding time.

#### 2.3.2. Chloride Erosion Depth

**Figure 3:**
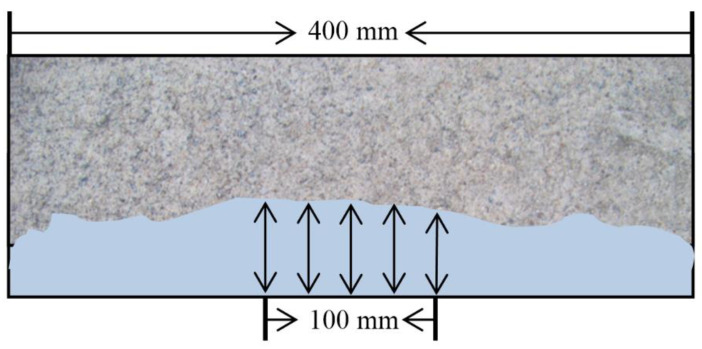
Sketch of the silver nitrate colorimetric method (depth measurement).

The corroded specimens were cut along the direction of the chloride ion erosion. 0.l mol/L silver nitrate solution was sprayed on the cross section of the specimens. A clear brown and white boundary was formed after the silver nitrate solution was sprayed. It was generally considered that the white area was the chloride erosion area and the brown area was the chloride-free area. The chloride erosion depth is shown in Figure 3. The average value of the five discoloration depths was considered as the chloride penetration depth of the concrete.

#### 2.3.3. Apparent Diffusion Coefficient (*D*_app_) and Apparent Surface Chloride Ion Concentration (*C*_s_)

The surface chloride ion concentration is not a fixed value and will accumulate and gradually reach stability with the extension of exposure erosion time. Wang et al. applied Fick’s diffusion law to perform a regression analysis on the experimental data and determined the diffusion coefficient and surface chloride ion concentration [24]. Researchers have studied the time-varying model of the chloride ion concentration on the concrete surface. Song et al. found that the time-varying law of the surface chloride ion concentration conforms to the power function model [25]. Kassir et al. found that the exponential model was more consistent with the time-varying law of surface chloride ion concentration [26]. In this study, Fick’s law was adopted to describe the chloride ion concentration at different depths for the concrete near the exposed surface. 

A regression analysis of the experimental data on the chloride ion concentrations at different depths of concrete under different conditions was carried out by applying Fick’s law of diffusion. The apparent surface chloride ion concentration and apparent diffusion coefficient were obtained at different erosion ages. The regression analysis was based on Equation (1):

(1) $$C=C_{s}\cdot \left(1-erf\frac{x}{2\sqrt{D_{\mathrm{app}}t}}\right)$$

where *C* is the measured chloride ion concentration at depth *x*; the fitting parameters are *C*_s_ and *D*_app_, based on the time-chloride penetration depth curve for each group.

### 2.4. Effect Coefficient K

Existences of sulfate ion and cyclic load have certain influence on the resistance of concrete to the chloride ion penetration. The influence can be expressed by the influence effect Coefficient *K*. Coefficient *K* was calculated in Equation (2):

*K* = *D*_x_/*D*_tm_ (2)

where *D*_x_ is the chloride ion *D*_app_ of concrete at a certain erosion age under the multifactor coupling action; *D*_tm_ is the chloride ion *D*_app_ of concrete at the same age under the conditions of chloride immersion. From the equation, it can be seen that a *K* value over 1.0 indicates that the sulfate ion or/and cyclic load is facilitating chloride ion diffusion.

## 3. Results and Discussion

### 3.1. Concentration Distribution of Chloride Ion

**Figure 4:**
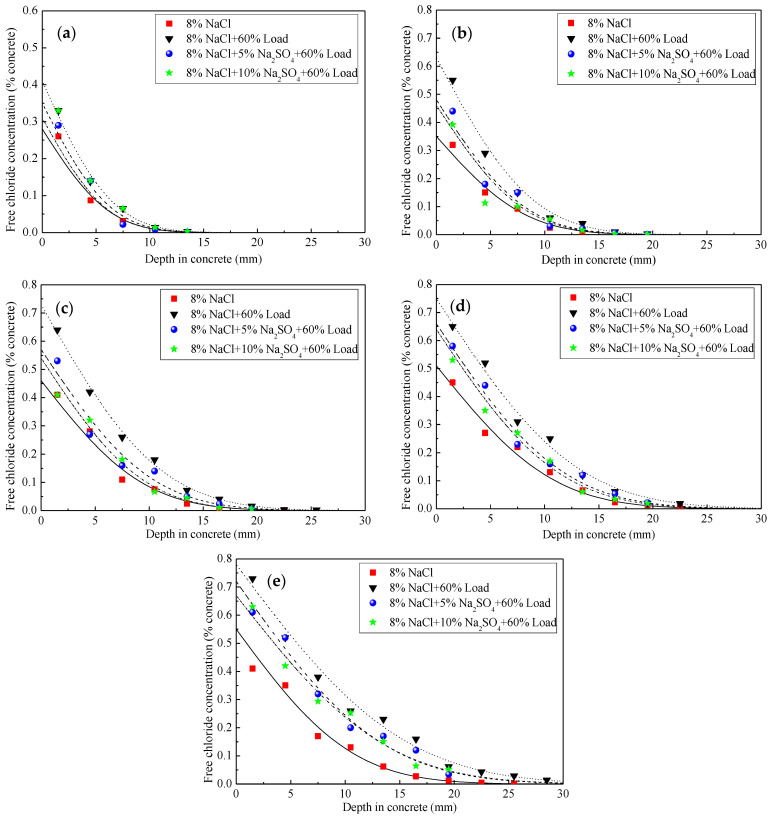
Chloride concentration distribution of concrete immersed in different sodium sulfate and sodium chloride coupling of cyclic loading condition. (**a**) 20 d; (**b**) 40 d; (**c**) 60 d; (**d**) 80 d; (**e**) 100 d.

The existence of load and sulfate ion did not change the general rule of chloride ion distribution in concrete, and the distribution of chloride ion concentration in concrete still complies with Fick’s second law (Figure 4). The chloride ion concentration at different depths under cyclic loading increased significantly compared with the immersion test. At a depth of 4.5 mm, the concentration of chloride was increased from 1.55 to 1.93 times while at the depth of 7.5 mm, the increase was between 1.66 to 2.11 times. The concentration of the chloride ion (100 d) at the depth of 7.5 mm was about 2.11 times of that without loading.

The concentration of chloride ions was relatively high under the load and dual-salt condition but less than the concentration under the coupling action of cyclic load and immersion. With the introduction of sulfate ions, the chloride ion concentration of each layer in the concrete decreases correspondingly with the increase of the sulfate solution concentration. When the erosion age was relatively short, the presence of sulfate ions in the dual-salt solution will make the internal pores more compact, thereby reducing the cyclic bending load to a certain extent on the promotion of the diffusion of chloride ions in the concrete [21].

### 3.2. The Time-Varying Law of C_*s*_ and D_*app*_

#### 3.2.1. Apparent Surface Chloride Ion Concentration (*C*_s_)

**Figure 5:**
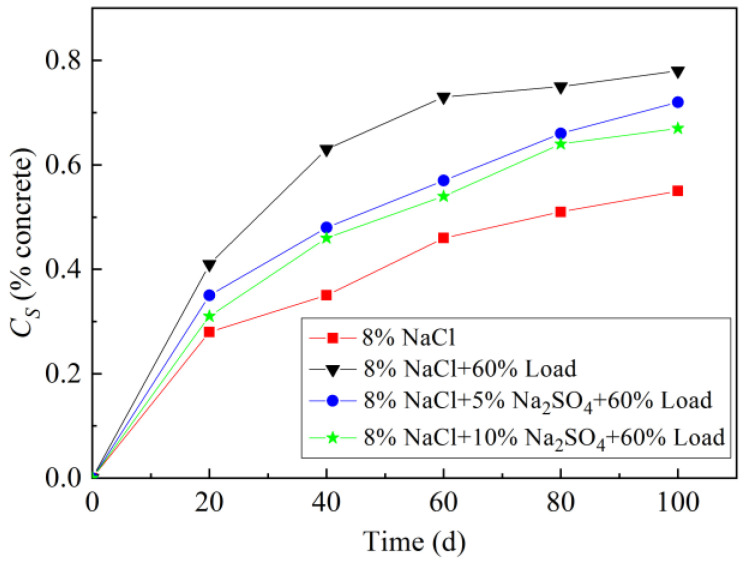
*C*_s_ of concrete immersed in different sodium sulfate and sodium chloride coupling of the cyclic loading condition.

Concrete *C*_s_ increases continuously with the extended erosion age (Figure 5). *C*_s_ (100 d) at a 60% stress level loading increased by 41.8%. The presence of sulfate did not change the overall time-varying characteristics of *C*_s_. *C*_s_ decreases due to the introduction of the sulfate ion, and its value decreases with the increase of sulfate concentration. The exponential function (Equation (3)) was used to fit *C*_s_ at different erosion ages, and the results are shown in Table 4.

*C*_s_ = *C*_smax_ × (1 − e^−*bt*^) (3)

where *t* is the soaking time (d); *C*_smax_ is the maximum *C*_s_; *b* is the accumulation rate. In this equation, two fitting parameters are *C*_smax_ and *b*, which is a typical mathematic description of the apparent surface chloride ion concentration.

Coefficients of determination (R^2^) in all fittings are higher than 0.95 (as shown in Table 4), indicating a satisfactory fitting accuracy. The presence of sulfate ions makes the surface chloride ion stability value *C*_smax_ and accumulation rate b decrease. *C*_smax_ and accumulation rate *b* gradually decrease with the increase of sulfate solution concentration.

#### 3.2.2. Apparent Diffusion Coefficient (*D*_app_)

**Figure 6:**
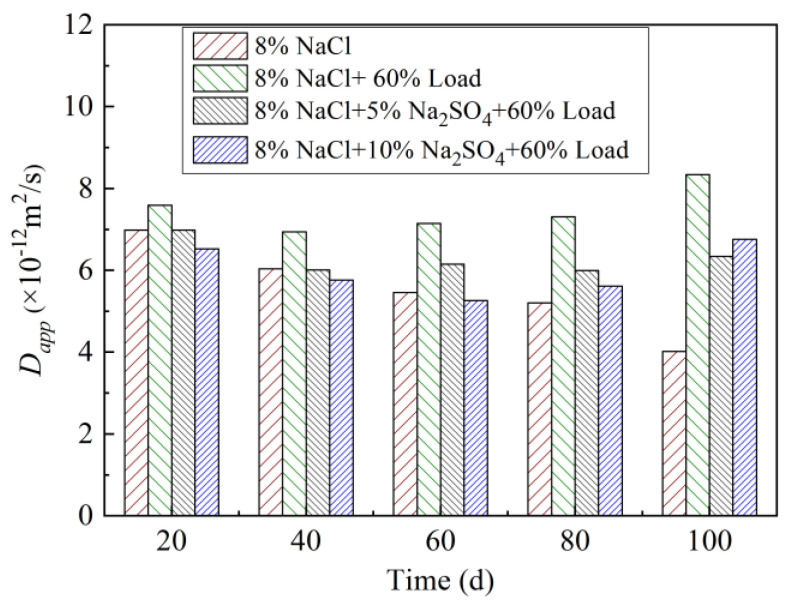
*D*_app_ of concrete immersed in different sodium sulfate and sodium chloride coupling of the cyclic loading condition.

Concrete *D*_app_ decreases first and then increases with the increase of the loading erosion age (Figure 6). Compared with the coupling effect of loading and single chloride salt, the *D*_app_ of concrete decreased gradually with the addition of sulfate before 80 d and decreased gradually with the increase of the sulfate ion concentration. When the erosion age is 100 d, *D*_app_ of 10% Na_2_SO_4_ test is slightly greater than that of 5%. When the erosion age is less than 80 d, the existence of sulfate ion will make the concrete structure denser, which delays the deterioration of concrete. When the erosion age is 100 d, with the high sulfate concentration, more crystallization products are generated; this leads to the internal cracking of concrete, which is manifested by the larger *D*_app_ when the sulfate concentration is high.

### 3.3. Chloride Erosion Depth

As shown in Table 5, the average and maximum chloride erosion depth increased significantly under the cyclic load, and the average depth increased by 49.3% with a 60% stress level compared with that without the load. The chloride erosion depth under the load reduced with the existence of the sulfate ion, and the higher the sulfate concentration was, the more obvious the depth reduction was. Under a 60% stress level load, the average chloride erosion depth of 5% Na_2_SO_4_ and 10% Na_2_SO_4_ decreased by 15.2% and 23.1%, respectively, while the maximum chloride erosion depth decreased by 2.8% and 16.9%. It was proved that the existence of sulfate can inhibit the chloride ion transport in concrete under cyclic loading in a short time.

### 3.4. Effect Coefficient K

**Figure 7:**
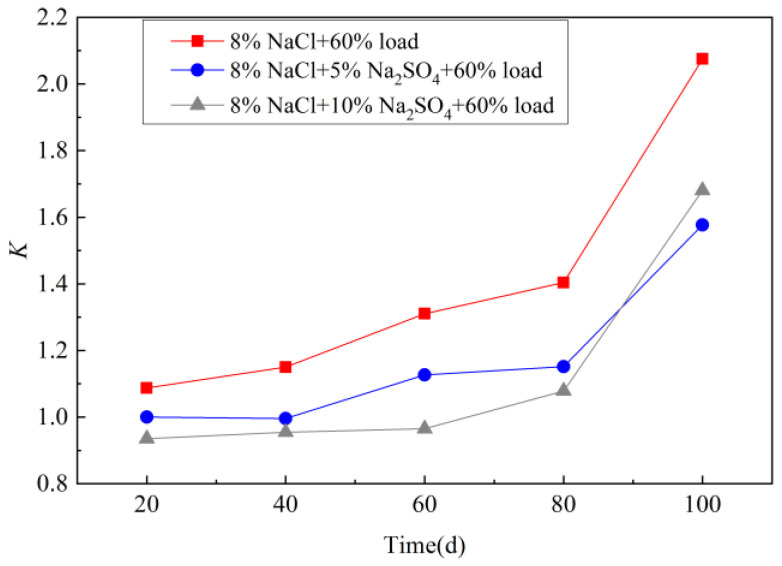
Regular time varying of the effect coefficient *K* immersed in different sodium sulfate and sodium chloride coupling of the cyclic loading condition.

The concrete effect coefficient *K* increases with the extension of the erosion age (Figure 7). Under the action of the 60% cyclic bending load, the *K* value is always greater than 1, which promotes chloride ion transport. Before 80 d, the *K* value decreased with the introduction of sulfate. The higher the sulfate concentration, the lower the *K* value was. When the erosion age was 100 d, the *K* value under the cyclic load of 10% sodium sulfate exceeds the concentration of 5%. It was caused by the combination of the compactness of sulfate crystallization products and cyclic load damage. Before 80 d, the interaction between the two was manifested as the compaction of sulfate crystal products, and the higher the concentration, the more promoted effect. As the concentration increased, the rate of crystallization product formation increased as well. This gradually changed the filling effect to expansive pressure induced by salt crystallization in confinement, which led to the increase of the concrete *K* value. At 100 d, the existence of sulfate crystals still played a filling role in general, so the *K* value was lower than that under the single cyclic load.

## 4. Conclusions

A testing scheme was designed and customized to test concrete samples under a cyclic load simultaneously coupled with a dual-salt ingress. The following conclusions can be drawn from the testing results:

- The long-term distribution of the chloride ion in the concrete immersed in sodium sulfate and sodium chloride solutions coupled with the cyclic load is, as expected, in accordance with Fick’s second law.
- Compared with the results of the single immersion test, the existence of the cyclic load significantly reduces the chloride erosion resistance of concrete. When the erosion age was 100 d, the chloride ion concentration at the depth of 7.5 mm was about 2.11 times that of the single immersion.
- In a certain erosion time, the existence of sulfate ion slowed down the transport of chloride ions. The depth of erosion (*C*_smax_) and apparent diffusion coefficient (*D*_app)_ of concrete decreased with the increase of the sulfate solution concentration. Under the condition of the dual-salt immersion and loading, the *K* value decreased continuously with the increase of erosion age and was less than 1, indicating that the existence of sulfate ion improved the resistance of concrete to chloride ion penetration.

## Figures and Tables

**Table 1:** Chemical composition of the cement.

Items	CaO	Al_2_O_3_	SiO_2_	Fe_2_O_3_	MgO	Alkali	Insoluble	Loss on Ignition
Weight (%)	64.98	5.33	21.69	3.47	1.17	1.04	0.07	2.68

**Table 2:** Proportion and flexural strength of the control samples.

W/C	Constituent (kg/m^3^)				Water Reducer (%)	Slump (mm)	28d Flexural Strength (MPa)
	Cement	Water	Fine Aggregate	Coarse Aggregate			
0.38	450	180	569	1211	0.3	40	6.87

**Table 3:** Experimental program of the concrete chloride ion erosion under the coupling condition of the cyclic load and dual salt.

Group	NaCl Concentration (%)	Na_2_SO_4_ Concentration (%)	Dynamic Loading Level		Loading Frequency (Hz)	Time (d)
			*S_min_*	*S_max_*		
1	8	0	0	0	0	20–100
2	8	0	10%	60%	0.02	20–100
3	8	5	10%	60%	0.02	20–100
4	8	10	10%	60%	0.02	20–100

**Table 4:** C_s_ exponential model fitting experimental results.

Experimental Condition	*C*_smax_	σ_2_/*C*_smax_	*b*	σ_2_/*b*	R^2^
8% NaCl	0.57520	0.03566	0.02744	0.00448	0.93388
8% NaCl + 60% Load	0.80118	0.01090	0.03740	0.00169	0.97870
8% NaCl + 5% Na_2_SO_4_ + 60% Load	0.74761	0.03885	0.02710	0.00368	0.95134
8% NaCl + 10% Na_2_SO_4_ + 60% Load	0.71781	0.02676	0.02597	0.00245	0.98098

**Table 5:** The erosion depth of concrete immersed in different sodium sulfate and sodium chloride coupling of the cyclic loading condition.

Experimental Condition	Measuring Point					Average Depth (mm)	Maximum Depth (mm)
	1	2	3	4	5		
8% NaCl	15.9	18.6	14.1	19.6	14.8	16.59	19.6
8% NaCl + 60% Load	25.4	19.9	26.6	24.5	26.9	24.67	26.9
8% NaCl + 5%Na_2_SO_4_ + 60% Load	19.8	17.9	22.7	24.1	20.2	20.92	24.1
8% NaCl + 10% Na_2_SO_4_ + 60% Load	15.4	22.3	19.8	20.0	17.2	18.96	22.3

## Data Availability

Not applicable.
